# Supplementation of dietary *Angelica sinensis* extracts to lactating Wuzhishan sows: effects on milk composition, immune function, milk-derived hormones, and related gene expression

**DOI:** 10.3389/fvets.2025.1524258

**Published:** 2025-05-15

**Authors:** Hongzhi Wu, Xilong Yu, Xiaoyu Zhang, Weiqi Peng, Fengjie Ji, Qian Shen, Renlong Lv

**Affiliations:** ^1^Tropical Crops Genetic Resources Research Institute, Chinese Academy of Tropical Agricultural Sciences, Haikou, China; ^2^College of Animal Science and Technology, Northeast Agricultural University, Harbin, China; ^3^College of Animal Science and Technology, Henan University of Science and Technology, Luoyang, China; ^4^Hainan Xuhuai Technology Co. Ltd., Haikou, China

**Keywords:** *Angelica sinensis* extracts, lactating sows, colostrum composition, immune function, milk hormone levels

## Abstract

The milk provided by sows plays an essential role in the growth and development of the piglets. This study aimed to explore the effects of *Angelica sinensis* extracts on milk composition, immune function, milk-derived hormones, and related gene expression of lactating sows. Forty-eight sows were randomly allocated into four groups, with six replicates and two sows per replicate. The control group (CON) was fed basal experimental diets, and the experimental groups were fed 400, 600, and 800 mg/kg *Angelica sinensis* extracts in powder (AS1, AS2, and AS3, respectively). Compared to the CON, (1) the lactoprotein and milk fat contents were higher (*p* < 0.05) in AS1, AS2, and AS3 at 12 and 24 h, and on the 7th day; (2) the IgA, IgG, and IL-2 levels were higher (*p* < 0.05), and the TNF-*α* levels were lower (*p* < 0.05) in AS1, AS2, and AS3 at 12 and 24 h; the IgG, IgM, and IL-2 levels were higher (*p* < 0.05) in AS1, AS2, and AS3 on the 7th day; (3) the progesterone, prolactin, growth hormone, and insulin-like growth factor-1 contents were higher (*p* < 0.05) in AS1, AS2, and AS3 at 12 and 24 h; the growth hormone and insulin-like growth factor-1 contents were higher (*p* < 0.05) in AS2 and AS3 on the 7th day; (4) the relative expressions of *PRLP*, *LALBA*, *AKT1*, *FASN*, *GLUT1,* and *CSN2* in mammary tissue were higher (*p* < 0.05) in groups treated with *Angelica sinensis* extracts; (5) the serum IgA, IgM, IL-6, growth hormone, insulin, and insulin-like growth factor-1 contents were higher (*p* < 0.05) in AS2 and AS3 in piglets; and (6) the relative expressions of *PRLP* and *IL-10* in umbilical cord were higher (*p* < 0.05) in groups treated with *Angelica sinensis* extracts. Incorporating 600 mg/kg of *Angelica sinensis* extracts into the diets of lactating Wuzhishan sows elicited differential responses regarding milk composition, immune function, hormonal profiles, and associated gene expression across distinct postpartum periods.

## 1 Introduction

The quality of the sow’s milk largely determines the growth and survival rates of the piglets it feeds (1, 2). The immunoglobulin fractions present in colostrum, particularly secretory IgA and IgG subclasses, confer passive humoral immunity to neonatal swine through pathogen-neutralizing mechanisms. At the same time, the multifaceted bioactive components of mature lactation, including oligosaccharides, lactoferrin, and growth factors, orchestrate gastrointestinal ontogeny by enhancing intestinal barrier function, modulating immune homeostasis, and facilitating the establishment of commensal microbiota colonization resistance (3–5). In addition, colostrum is rich in lactose and lipids, which provide newborn piglets with the necessary energy to maintain their thermal homeostasis (5). Periparturient dietary supplementation with immunonutritional agents, including conjugated linoleic acid (CLA), marine-derived omega-3 polyunsaturated fatty acids (n-3 PUFAs), phytogenic bioactive compounds, probiotic microbial consortia, and yeast-derived mannoprotein complexes, has been shown to epigenetically modulate lactogenic programming in sows, resulting in quantifiable enhancements of mammary secretory output through increased immunoglobulin titers, optimized fatty acid profiles, and amplified synthesis of antimicrobial factors in colostrum and transitional milk (6, 7).

*Angelica sinensis* is a commonly used Chinese herbal medicine, known for its effects in tonifying and activating blood, regulating menstruation, relieving pain, enhancing immunity, reducing inflammation, providing analgesic effects, acting as a laxative, and promoting digestion (8, 9). Zhao et al. found that *Angelica sinensis* improved placental structure, attenuated inflammatory response, and modulated placental angiogenesis and growth factor receptors in sows (10). Wu et al. reported that *Angelica sinensis* improved the intestinal floras and short-chain fatty acids of cecal contents in laying chickens (11). Tian et al. found that *Angelica sinensis* polysaccharide attenuated LPS-induced inflammation response of primary dairy cow claw dermal cells via nuclear factor kappa-B (NF-κB) and mitogen-activated protein kinase (MAPK) signaling pathways (12). Duan et al. found that Chinese herbal complexes containing *Angelica sinensis* added to the periparturient sow diet significantly augmented antioxidant capacity in both sows and offspring piglets (13). Currently, there are relatively few studies related to the study of *Angelica sinensis* on milk composition and immune and hormonal indicators in milk of local sows. This study posits that dietary supplementation with *Angelica sinensis* extracts enhances lactogenic performance in Wuzhishan sows through dose-dependent modulation of milk composition, immune function, milk-derived hormones, and related gene expression.

The Wuzhishan pig is a small breed native to the Wuzhishan region of Hainan Province in China (14). It is characterized by a compact, firm body, a small and slightly long head, a straight and long snout, a pointed mouth, and a prominent muzzle protrusion. The pig also has small, thin ears, a peach-shaped appearance close to the neck, and a rat-like head (15, 16). The objective of this study was to (a) explore the effects of *Angelica sinensis* extracts on the milk composition, immune function, milk-derived hormones, and related gene expression of lactating sows; (b) conduct a preliminary investigation of the action mechanism of *Angelica sinensis* extracts on the lactation ability of sows; and (c) explore the valuable addition of *Angelica sinensis* extracts in the feed of gestating and lactating sows.

## 2 Materials and methods

### 2.1 Experiment material

Gestating and lactating Wuzhishan sows, with an average body weight of 33.00 ± 2.00 kg, were primiparous, had the same genetic background, and shared similar birthing date.

*Angelica sinensis* (AS) extracts were purchased from Shaanxi Baichuan Biotechnology Co., Ltd. The active ingredient contents of the AS extracts were tested by the liquid chromatograph and mass spectrometer. The *Angelica sinensis* polysaccharide contents of AS extracts were 12.45%, and the ferulic acid content was 0.06%.

Milk immunoglobulin and serum biochemical kits were purchased from Shanghai Sangon Biotechnology Co., Ltd., Shanghai, China.

### 2.2 Experiment design and sample collection

The added amount of *Angelica sinensis* extract was a single influencing factor in this study. Forty-eight Wuzhishan sows were randomly allocated into four groups, with six replicates and two sows per replicate. The basal experimental diets for gestating and lactating were corn-soybean meal-type diets formulated according to the NCR (2012) standards. The composition of the basal experimental diets and nutritional levels is shown in Table 1. The control group (CON) was fed basal experimental diets, and the experimental groups were fed 400, 600, and 800 mg/kg *Angelica sinensis* extracts in powder form (AS1, AS2, and AS3, respectively) according to recommended usage. The experiment started on day 107 of gestation and ended on day 7 after delivery. Sows were guaranteed free access to feed and water during the trial.

**Table 1:** Composition (kg/100 kg) of the basal experimental diets^1^ for gestating and lactating sows.

Items	Gestation period	Lactating period
Ingredients		
Corn, %	64.00	59.20
Soybean meal, %	23.24	26.90
Fish meal, %	2.00	2.00
Soybean oil, %	2.00	4.00
Wheat bran, %	4.00	3.00
Calcium hydrogen phosphate, %	1.85	1.88
Calcium carbonate, %	0.97	0.92
Salt, %	0.40	0.40
Sodium hydrogen carbonate, %	0.40	0.39
L-Lysine hydrochloride, %	-	0.24
Premix^2^, %	1.14	1.07
Nutrient levels, on an air-dry basis		
Digestible energy^3^, MJ/kg	13.37	13.81
Crude protein^4^, %	17.50	18.50
Calcium^4^, %	0.95	0.98
Phosphorus^4^, %	0.75	0.81
Available phosphorus^4^, %	0.45	0.47
Total lysine^4^, %	0.85	1.12

1 Based on the NRC (2012) nutrient requirements for gestation and lactating sows.

2 The premix provided the following per kg of diet: VA 2000 IU, VD 200 IU, VE 45 IU, VK 0.5 mg, VB1 1 mg, pantothenic acid 12 mg, nicotinic acid 10.25 mg, VB6 3.85 mg, VB12 15 μg, folic acid 1.35 mg, biotin 0.21 mg, VC 200 mg, Mn as manganese sulfate 20 mg, Fe as ferrous sulfate 80 mg, Cu as copper sulfate 5 mg, I as potassium iodide 0.14 mg, and Se as sodium selenite 0.15 mg.

3 Calculated value (NRC, 2012).

4 Analyzed content.

Approximately 30 ml of colostrum were collected from the front, middle, and rear nipples of the sows in the 0, 12, and 24 h and on the 7th day after the farrowing ended and then mixed well and stored at –20°C until further testing. Four milliliters of lidocaine hydrochloride were injected into the third left papilla of the sows for local anesthesia. A total of 2–4 g of mammary tissue samples were collected in a 2 ml cryopreservative tube using a live sampling gun (BARD® MAGNUM®, MG1522, USA) and disposable sampling needles (C.R. Bard. Inc., Covington, GA, USA) after the farrowing ended, and approximately 2 cm of the umbilical cord was collected approximately 6–8 cm from the piglet’s abdomen. The mammary and umbilical cord tissue samples were stored at −80°C for further use. After birth, two piglets per replicate were randomly selected for approximately 3 ml of marginal ear vein blood collection. After resting the blood for 15 min, it was centrifuged at 3000 rpm for 15 min to obtain the serum, which was divided into Eppendorf tubes and stored at −20°C for testing.

### 2.3 Milk components

The lactoprotein content, lactose, milk fat, and solids-not-fat in milk were quantified via Fourier-transform near-infrared spectroscopy using a commercially calibrated Milk-Scan 134 A/B system (Beijing LiO Biotechnology Co., China) with wavelength calibration against bovine milk reference standards (ISO 9622:2013). Pre-analytical processing involved refrigerated differential centrifugation (3,000 × *g*, 20 min, 4°C) to remove cellular debris and lipid stratification prior to spectral acquisition in triplicate (*λ* = 850–1,050 nm, resolution 8 cm^−1^).

### 2.4 Immune function and milk-derived hormone levels

Serum immunophenotypic parameters (IgA, IgG, and IgM), pro−/anti-inflammatory cytokines (IL-2, IL-6, and TNF-α), and metabolic hormones, including growth hormone (GH), insulin, and insulin-like growth factor-1 (IGF-1), were quantified via standardized enzyme-linked immunosorbent assays (ELISAs) using commercially available kits (Shanghai Sangon Biotechnology Co., Ltd., China), with all procedures conducted in triplicate under quality-controlled conditions (inter-assay CV < 8.0%, intra-assay CV < 5.0%) according to ISO 17025-certified manufacturer protocols (Cat. Nos. SS-ELISA-001 to 009).

### 2.5 RNA extraction and quantitative analysis of mRNA with real-time PCR

The mammary and umbilical cord tissue samples of soybean size were taken, and RNA was extracted according to the instructions of the animal tissue RNA extraction kit. The quality of RNA was assessed by a 2% agarose gel electrophoresis. An ultra-microspectrophotometer (NanoPhotometer, Implen, Germany) was used to evaluate the total RNA concentration and purity (A260/A280 ratio). The primer sequences are shown in Table 2, and the primers corresponding to the sow gene sequence were synthesized using Sangon (Shanghai, China). The cDNA was synthesized using the PrimeScript^®^ RT regent kit with the gDNA Eraser (TaKaRa, Dalian, China) reverse transcription kit. DNA was removed from the sample using a reaction system as 2-μl 5 × gDNA Eraser Buffer, 1 μl gDNA Eraser, and 1 μg RNA and then replenished the volume to 10 μl of RNase Free ddH_2_O (1). The system of reverse transcription was 10 μl reaction liquid, 1 μl RT Primer Mix, 4 μl RNase-free ddH_2_O, 4-μl 5 × PrimeScript^®^ Buffer 2, and PrimeScript^®^ RT Enzyme MixI. The reaction procedure was maintained at 37°C constant temperature for 15 min, 85°C constant temperature for 5 s, and 4°C stored briefly. The obtained cDNA was stored at −20°C (17). The housekeeping gene of *glyceraldehyde-3-phosphate dehydrogenase* (*GAPDH*) was used. The relative mRNA levels of *prolactin receptor* (*PRLP*), *alpha-lactalbumin* (*LALBA*), *serine/threonine kinases* (*AKT1*), *beta-1,4-galactosyltransferase 1* (*β4GALT1*), *fatty acid synthase* (*FASN*), *glucose transporter* (*GLUT1*), *beta-casein* (*CSN2*), *interleukin-10* (*IL-10*), and *tumor necrosis factor-α* (*TNF-α*) were calculated using the 2^-ΔΔCt^ method.

**Table 2:** Primer sequence list.

Gene	Gene name	Forward and reverse primers	Product size	Accession No.
PRLP	Prolactin receptor	F: 5′-GGCTCCGTTTGAAGAACCAA-3′	67	NM_001001868.1
		R: 5′-GTCTTTCGCAGCTGGATTCTG-3′		
LALBA	Alpha-Lactalbumin	F: 5′-GTGGTGGGGATTCTCTTTCC-3′	179	NM_214360
		R: 5′-TCTGTGCTGCCATTGTCATG-3′		
AKT1	Serine/Threonine kinases	F: 5′-CCTGAAGAAGGAGGTCATCG-3′	81	NM_001159776
		R: 5′-TCGTGGGTCTGGAAGGAGTA-3′		
β4GALT1	Beta-1,4-galactosyltransferase 1	F: 5′-GAGTTTAACATGGCGTGGAC-3′	185	XM003130680
		R: 5′-TGACGCTGTAGGATTGGGTG-3′		
FASN	Fatty acid synthase	F: 5′-GCTTGTCCTGGGAAGAGTGTA-3′	115	NM001099930
		R: 5′-AGGAACTCGGACATAGCGG-3′		
GLUT1	Glucose transporter	F: 5′-GATGAAGGAGGAGTGCCG-3′	106	EU012358
		R: 5′-CAGCACCACGGCGATGAGGAT-3′		
CSN2	Beta-casein	F: 5′-GGACTTGATCGCCATGAAGCT-3′	81	NM_214434.2
		R: 5′-GCATTGAGTTCTTCCTTCGCTCT-3′		
IL-10	Interleukin-10	F: 5′-GTGGCAGCCAGCATTAAGTC-3′	100	NM_214041.1
		R: 5′-AACTCTTCACTGGGCCGAAG-3′		
TNF-α	Tumor necrosis factor-α	F: 5′-CCAGACCAAGGTCAACCTCC-3′	89	NM_214022.10
		R: 5′-TCCCAGGTAGATGGGTTCGT-3′		
GAPDH	Glyceraldehyde-3-phosphate Dehydrogenase	F: 5′-GTCGGAGTGAACGGATTTGG-3′	76	NM_001206359.1
		R: 5′-CAATGTCCACTTTGCCAGAGTTAA-3′		

### 2.6 Statistical analysis

Statistical analyses were conducted using SPSS 29.0 statistics software (NY, USA). The Kolmogorov–Smirnov test was used to check if all data in this study followed a normal distribution. Data were expressed as mean ± SEM. Statistical comparisons of different treatments were performed using the one-way analysis of variance (ANOVA). The test results of all analyses were considered significant at a *p-*value of < 0.05.

## 3 Result

### 3.1 Effects of *Angelica sinensis* extracts on milk components of lactating sows

The lactoprotein and solids-not-fat contents in AS2 and AS3 were higher (*p* < 0.05) than in the CON at 0 h. The lactoprotein and milk fat contents were higher (*p* < 0.05) in groups treated with *Angelica sinensis* extracts than in the CON, and they were higher (*p* < 0.05) in AS2 and AS3 than in SA1 at 12 h. The lactoprotein, lactose, and milk fat contents were higher (*p* < 0.05) in groups treated with *Angelica sinensis* extracts compared to the CON, and the lactoprotein and milk fat contents were higher (*p* < 0.05) in AS2 and AS3 than in AS1 at 24 h. The lactoprotein, milk fat, and solids-not-fat contents were higher (*p* < 0.05) in groups treated with *Angelica sinensis* extracts than in the CON, and the lactoprotein contents were higher (*p* < 0.05) in AS2 and AS3 than in AS1 on the 7th day after delivery (Table 3).

**Table 3:** Effects of Angelica sinensis extracts on milk components of lactating sows.

Items	CON	AS1	AS2	AS3	*p*-value
0 h					
Lactoprotein, %	11.89 ± 0.21^b^	12.01 ± 0.21^b^	13.56 ± 0.27^a^	13.68 ± 0.30^a^	0.0237
Lactose, %	3.12 ± 0.03	3.14 ± 0.04	3.11 ± 0.03	3.16 ± 0.04	0.6217
Milk fat, %	2.61 ± 0.21	2.75 ± 0.27	2.81 ± 0.30	2.82 ± 0.29	0.0689
Solids-not-fat, %	19.81 ± 0.82^c^	22.01 ± 0.15^b^	24.15 ± 0.27^a^	24.96 ± 0.33^a^	0.0361
12 h					
Lactoprotein, %	19.87 ± 0.34^c^	21.92 ± 0.23^b^	23.57 ± 0.19^a^	23.46 ± 0.21^a^	0.0452
Lactose, %	3.61 ± 0.21	3.59 ± 0.12	3.78 ± 0.19	3.69 ± 0.26	0.5665
Milk fat, %	4.82 ± 0.16^c^	5.12 ± 0.09^b^	5.56 ± 0.12^a^	5.61 ± 0.16^a^	0.0481
Solids-not-fat, %	21.52 ± 0.56	22.23 ± 0.21	23.15 ± 0.25	23.22 ± 0.27	0.0557
24 h					
Lactoprotein, %	20.12 ± 0.22^c^	23.52 ± 0.12^b^	25.12 ± 0.10^a^	25.31 ± 0.16^a^	0.0321
Lactose, %	3.31 ± 0.12^b^	3.70 ± 0.11^a^	3.72 ± 0.16^a^	3.76 ± 0.11^a^	0.0212
Milk fat, %	4.92 ± 0.09^d^	5.21 ± 0.10^c^	5.49 ± 0.11^b^	5.70 ± 0.12^a^	0.0325
Solids-not-fat, %	22.12 ± 0.42	23.11 ± 0.23	23.12 ± 0.25	23.22 ± 0.21	0.6245
7 days					
Lactoprotein, %	8.23 ± 0.12^c^	8.55 ± 0.06^b^	8.96 ± 0.11^a^	9.02 ± 0.11^a^	0.0212
Lactose, %	4.00 ± 0.25	4.12 ± 0.19	4.11 ± 0.21	4.09 ± 0.15	0.0625
Milk fat, %	6.62 ± 0.12^b^	7.11 ± 0.19^a^	7.12 ± 0.25^a^	7.06 ± 0.30^a^	0.0567
Solids-not-fat, %	18.26 ± 0.15^b^	20.12 ± 0.19^a^	21.32 ± 0.21^a^	21.45 ± 0.29^a^	0.0456

^a,b,c,d^Values with different small letter superscripts in the same column mean a significant difference (*p* < 0.05).

### 3.2 Effects of *Angelica sinensis* extracts on milk immune indexes of lactating sows

The IgA and IL-2 levels were higher (*p* < 0.05) in the groups treated with *Angelica sinensis* extracts, the IgM levels were higher (*p* < 0.05) in AS2 and AS3, and the TNF-*α* levels were lower (*p* < 0.05) in AS1, AS2, and AS3 compared to the CON at 0 h. The IgA, IgG, and IL-2 levels were higher (*p* < 0.05) in AS1, AS2, and AS3, the IgM levels were higher (*p* < 0.05) in AS2 and AS3, the TNF-*α* levels were lower (*p* < 0.05) in groups treated with *Angelica sinensis* extracts compared to the CON, and the IgA, IgM, and IL-2 levels were higher (*p* < 0.05) in AS2 and AS3 than in AS1 at 12 h. The IgA, IgG, IgM, and IL-2 levels were higher (*p* < 0.05), the TNF-α levels were lower (*p* < 0.05) in groups treated with *Angelica sinensis* extracts compared to the CON, and the IgA and IgG levels were higher (*p* < 0.05) in AS2 and AS3 than in AS1 at 24 h. The IgG, IgM, and IL-2 levels were higher (*p* < 0.05) in AS1, AS2, and AS3 compared to the CON, and the IgG levels were higher (*p* < 0.05) in AS2 and AS3 than in AS1 on the 7th day after delivery (Table 4).

**Table 4:** Effects of Angelica sinensis extracts on milk immune indexes of lactating sows.

Items	CON	AS1	AS2	AS3	*P*-value
0 h					
IgA, μg/ml	1.67 ± 0.03^b^	1.82 ± 0.06^a^	1.85 ± 0.09^a^	1.84 ± 0.10^a^	0.0345
IgG, μg/ml	18.25 ± 0.25	18.36 ± 0.31	18.67 ± 0.29	18.66 ± 0.35	0.3325
IgM, μg/ml	1.77 ± 0.11^b^	1.98 ± 0.12^ab^	2.21 ± 0.15^a^	2.22 ± 0.13^a^	0.0456
IL-2, ng/L	300 ± 5.21^b^	315 ± 6.24^a^	319 ± 5.46^a^	317 ± 5.28^a^	0.0389
IL-6, ng/L	78.12 ± 2.23	80.15 ± 3.56	80.55 ± 3.24	79.25 ± 3.33	0.0659
TNF-α, ng/L	280 ± 2.31^a^	270 ± 3.34^b^	271 ± 4.26^b^	273 ± 2.34^b^	0.0314
12 h					
IgA, μg/ml	1.87 ± 0.02^c^	2.08 ± 0.04^b^	2.21 ± 0.05^a^	2.21 ± 0.06^a^	0.0256
IgG, μg/ml	20.12 ± 0.25^b^	23.96 ± 0.24^a^	23.57 ± 0.22^a^	24.01 ± 0.27^a^	0.0354
IgM, μg/ml	2.00 ± 0.02^b^	2.00 ± 0.01^b^	2.11 ± 0.05^a^	2.12 ± 0.03^a^	0.0311
IL-2, ng/L	335 ± 4.98^c^	346 ± 2.38^b^	359 ± 2.56^a^	360 ± 3.97^a^	0.0268
IL-6, ng/L	60.25 ± 2.26	61.33 ± 3.65	59.36 ± 3.24	61.26 ± 3.59	0.1567
TNF-α, ng/L	300 ± 6.25^a^	280 ± 6.23^b^	286 ± 6.27^b^	279 ± 6.89^b^	0.0349
24 h					
IgA, μg/ml	1.90 ± 0.03^c^	1.99 ± 0.02^b^	2.12 ± 0.02^a^	2.14 ± 0.04^a^	0.0358
IgG, μg/ml	22.91 ± 0.15^c^	24.56 ± 0.11^b^	27.62 ± 0.10^a^	27.65 ± 0.13^a^	0.0256
IgM, μg/ml	2.03 ± 0.12^b^	2.23 ± 0.08^a^	2.20 ± 0.10^a^	2.26 ± 0.15^a^	0.0349
IL-2, ng/L	340 ± 2.68^b^	356 ± 3.54^a^	351 ± 3.20^a^	357 ± 3.59^a^	0.0468
IL-6, ng/L	50.25 ± 2.36	51.23 ± 3.64	52.16 ± 2.95	52.44 ± 3.67	0.1927
TNF-α, ng/L	276 ± 3.56^a^	260 ± 2.56^b^	262 ± 3.02^b^	265 ± 3.89^b^	0.0489
7 days					
IgA, μg/ml	1.43 ± 0.21	1.46 ± 0.19	1.51 ± 0.11	1.50 ± 0.14	0.0968
IgG, μg/ml	16.12 ± 0.11^b^	16.13 ± 0.12^b^	18.19 ± 0.15^a^	18.26 ± 0.17^a^	0.0325
IgM, μg/ml	1.50 ± 0.01^b^	1.62 ± 0.04^a^	1.60 ± 0.02^a^	1.61 ± 0.01^a^	0.0256
IL-2, ng/L	320 ± 6.26^b^	340 ± 5.29^a^	346 ± 6.82^a^	342 ± 6.97^a^	0.0355
IL-6, ng/L	49.56 ± 2.36	49.69 ± 3.26	50.26 ± 2.58	51.62 ± 3.65	0.3332
TNF-α, ng/L	260 ± 2.56	260 ± 3.56	266 ± 6.29	261 ± 4.25	0.3958

^a,b,c^Values with different small letter superscripts in the same column mean a significant difference (*p* < 0.05). IgA, immunoglobulin A; IgG, immunoglobulin G; IgM, immunoglobulin M; IL-2, interleukin-2; IL-6, interleukin-6; TNF-α, tumor necrosis factor-α.

### 3.3 Effects of *Angelica sinensis* extracts on milk hormone levels of lactating sows

The progesterone, prolactin, and insulin contents were higher (*p* < 0.05) in AS2 and AS3 than in the CON at 0 h. The progesterone, prolactin, growth hormone, and insulin-like growth factor-1 contents were higher (*p* < 0.05) in AS1, AS2, and AS3, the insulin contents were higher (*p* < 0.05) in AS2 and AS3 than in the CON, and the progesterone contents were higher (*p* < 0.05) in AS2 and AS3 than in AS1 at 12 h. The progesterone, prolactin, insulin, and insulin-like growth factor-1 contents were higher (*p* < 0.05) in groups treated with *Angelica sinensis* extracts than in the CON, and the prolactin, growth hormone, and insulin contents were higher (*p* < 0.05) in AS2 and AS3 than in AS1 at 24 h. The growth hormone and insulin-like growth factor-1 contents were higher (*p* < 0.05) in AS2 and AS3 compared to the CON, and the growth hormone contents were higher (*p* < 0.05) in AS2 and AS3 than in AS1 on the 7th day after delivery (Table 5).

**Table 5:** Effects of Angelica sinensis extracts on milk hormone levels of lactating sows.

Items	CON	AS1	AS2	AS3	*p*-value
0 h					
Progesterone, pmol/L	2,430 ± 10.25^c^	2,578 ± 10.36^b^	2,621 ± 20.15^a^	2,630 ± 16.34^a^	0.0356
Prolactin, ng/L	576 ± 10.25^b^	592 ± 13.24^b^	623 ± 10.25^a^	630 ± 15.23^a^	0.0422
Growth hormone, ng/ml	26.03 ± 1.25	26.59 ± 2.03	26.51 ± 2.06	26.33 ± 1.56	0.1258
Insulin, mlU/L	48.82 ± 1.26^b^	52.12 ± 1.36^ab^	53.12 ± 1.28^a^	53.56 ± 1.56^a^	0.0459
Insulin-like growth factor-1, μg/L	242 ± 10.25	250 ± 21.10	249 ± 16.35	245 ± 15.87	0.5217
12 h					
Progesterone, pmol/L	2,320 ± 11.56^c^	2,410 ± 15.69^b^	2,478 ± 12.31^a^	2,469 ± 15.23^a^	0.0421
Prolactin, ng/L	650 ± 11.25^b^	680 ± 12.25^a^	690 ± 16.23^a^	687 ± 14.23^a^	0.0325
Growth hormone, ng/ml	29.06 ± 1.02^b^	32.12 ± 1.04^a^	33.21 ± 1.24^a^	32.89 ± 1.29^a^	0.0356
Insulin, mlU/L	50.12 ± 1.59^b^	52.15 ± 1.54^ab^	55.53 ± 2.29^a^	56.12 ± 2.67^a^	0.0459
Insulin-like growth factor-1, μg/L	249 ± 10.26^b^	281 ± 9.26^a^	286 ± 10.36^a^	288 ± 10.33^a^	0.0388
24 h					
Progesterone, pmol/L	2,127 ± 20.56^b^	2,256 ± 19.96^a^	2,240 ± 29.30^a^	2,236 ± 28.46^a^	0.0238
Prolactin, ng/L	701 ± 10.26^c^	723 ± 12.25^b^	760 ± 10.24^a^	770 ± 15.26^a^	0.0359
Growth hormone, ng/ml	32.12 ± 1.26^b^	32.56 ± 2.00^b^	35.21 ± 1.56^a^	35.12 ± 1.23^a^	0.0459
Insulin, mlU/L	50.56 ± 2.01^c^	53.26 ± 1.56^b^	56.31 ± 1.26^a^	56.89 ± 1.28^a^	0.0358
Insulin-like growth factor-1, μg/L	260 ± 10.25^b^	290 ± 12.25^a^	295 ± 10.36^a^	289 ± 15.26^a^	0.0457
7 days					
Progesterone, pmol/L	2001 ± 20.56	1996 ± 27.56	2010 ± 30.56	2008 ± 28.97	0.0956
Prolactin, ng/L	710 ± 10.23	722 ± 15.23	725 ± 20.15	730 ± 19.30	0.0658
Growth hormone, ng/ml	33.26 ± 1.26^b^	33.45 ± 1.59^b^	37.89 ± 1.25^a^	37.90 ± 2.03^a^	0.0368
Insulin, mlU/L	55.36 ± 1.28	54.26 ± 2.05	55.55 ± 2.16	54.98 ± 1.57	0.5647
Insulin-like growth factor-1, μg/L	275 ± 10.21^b^	280 ± 12.56^ab^	300 ± 12.99^a^	312 ± 10.15^a^	0.0238

^a,b,c^Values with different small letter superscripts in the same column mean a significant difference (*p* < 0.05).

### 3.4 Effect of *Angelica sinensis* extracts on mammary tissue gene expression of lactating sows

**Figure 1:**
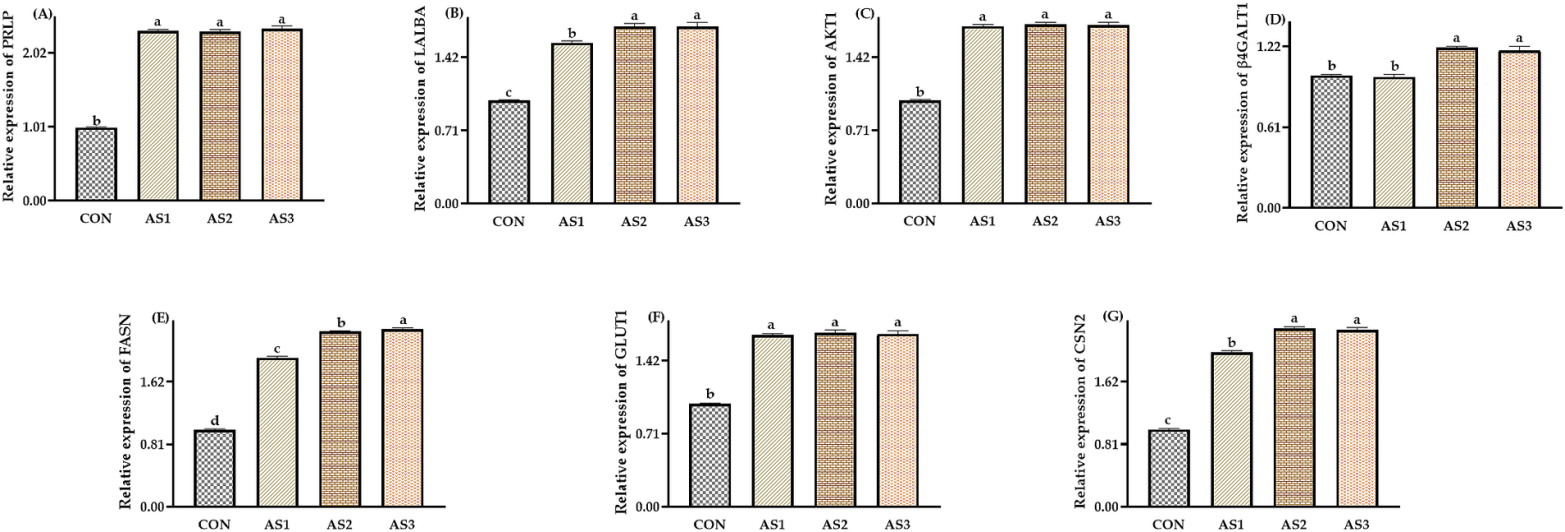
Effects of Angelica sinensis extract on mammary tissue gene expression of lactating sows. **(A)** The data of relative expression levels of *PRLP* (*prolactin receptor*) in the CON, AS1, AS2, and AS3 were 1.00 ± 0.01^b^, 2.32 ± 0.02^a^, 2.31 ± 0.02^a^, and 2.35 ± 0.04^a^, individually, *p* = 0.0207; **(B)** the data of relative expression levels of *LALBA* (*alpha-lactalbumin*) in the CON, AS1, AS2, and AS3 were 1.00 ± 0.01^c^, 1.56 ± 0.02^b^, 1.72 ± 0.03^a^, and 1.72 ± 0.04^a^, individually, *p* = 0.0397; **(C)** the data of relative expression levels of *AKT1* (*serine/threonine kinases*) in the CON, AS1, AS2, and AS3 were 1.00 ± 0.01^b^, 1.72 ± 0.02^a^, 1.74 ± 0.02^a^, and 1.73 ± 0.03^a^, individually, *p* = 0.0486; **(D)** the data of relative expression levels of *β4GALT1* (*beta-1,4-galactosyltransferase 1*) in the CON, AS1, AS2, and AS3 were 1.00 ± 0.01^b^, 0.99 ± 0.02^b^, 1.21 ± 0.01^a^, and 1.19 ± 0.03^a^, individually, *p* = 0.0424; **(E)** the data of relative expression levels of *FASN* (*fatty acid synthase*) in the CON, AS1, AS2, and AS3 were 1.00 ± 0.01^d^, 1.93 ± 0.02^c^, 2.27 ± 0.01^b^, and 2.30 ± 0.02^a^, individually, *p* = 0.0364; **(F)** the data of relative expression levels of *GLUT1* (*glucose transporter*) in the CON, AS1, AS2, and AS3 were 1.00 ± 0.01^b^, 1.67 ± 0.01^a^, 1.69 ± 0.03^a^, and 1.68 ± 0.03^a^, individually, *p* = 0.0268; **(G)** the data of relative expression levels of *CSN2* (*beta-casein*) in the CON, AS1, AS2, and AS3 were 1.00 ± 0.01^c^, 2.00 ± 0.02^b^, 2.31 ± 0.02^a^, and 2.29 ± 0.03^a^, individually, *p* = 0.0237. ^a,b,c,d^Values with different small letter superscripts in the same column mean a significant difference (*p* < 0.05).

The relative expressions of *PRLP*, *LALBA*, *AKT1*, *FASN*, *GLUT1,* and *CSN2* were higher (*p* < 0.05) in groups treated with *Angelica sinensis* extracts than in the CON, the relative expressions of *LALBA*, *FASN*, and *CSN2* were higher (*p* < 0.05) in AS2 and AS3 than in AS1, and the relative expressions of *FASN* were higher (*p* < 0.05) in AS3 than in AS2. The relative expressions of *β4GALT1* were higher (*p* < 0.05) in AS2 and AS3 than in the CON and AS1 (Figure 1).

### 3.5 Effects of *Angelica sinensis* extracts on immune indexes of piglets

The IgA, IgM, and IL-6 levels were higher (*p* < 0.05) in groups treated with *Angelica sinensis* extracts, the IL-2 levels were higher (*p* < 0.05) in AS2 and AS3 compared to the CON, and the IgM and IL-6 levels were higher (*p* < 0.05) in AS2 and AS3 than in AS1 in serum of piglets (Table 6).

**Table 6:** Effects of Angelica sinensis extracts on immune indexes of piglets.

Items	CON	AS1	AS2	AS3	*p*-value
IgA, μg/ml	0.86 ± 0.02^b^	1.02 ± 0.05^a^	1.06 ± 0.02^a^	1.08 ± 0.01^a^	0.0265
IgG, μg/ml	12.56 ± 1.02	13.52 ± 1.49	13.55 ± 1.48	13.57 ± 2.03	0.0986
IgM, μg/ml	2.01 ± 0.03^c^	2.15 ± 0.06^b^	2.36 ± 0.05^a^	2.39 ± 0.08^a^	0.0354
IL-2, ng/L	200 ± 10.21^b^	220 ± 15.36^ab^	259 ± 15.68^a^	264 ± 18.22^a^	0.0469
IL-6, ng/L	30.25 ± 1.26^c^	34.59 ± 1.55^b^	39.84 ± 1.29^a^	39.86 ± 1.49^a^	0.0359
TNF-α, ng/L	156 ± 5.69	150 ± 7.82	163 ± 10.55	160 ± 15.64	0.2597

^a,b,c^Values with different small letter superscripts in the same column mean a significant difference (*p* < 0.05). IgA, immunoglobulin A; IgG, immunoglobulin G; IgM, immunoglobulin M; IL-2, interleukin-2; IL-6, interleukin-6; TNF-α, tumor necrosis factor-α.

### 3.6 Effects of *Angelica sinensis* extracts on serum hormone levels of piglets

The growth hormone, insulin, and insulin-like growth factor-1 contents were higher (*p* < 0.05) in groups treated with *Angelica sinensis* extracts than in the CON, and the insulin and insulin-like growth factor-1 contents were higher (*p* < 0.05) in AS2 and AS3 than in AS1 in serum of piglets (Table 7).

**Table 7:** Effects of Angelica sinensis extracts on serum hormone levels of piglets.

Items	CON	AS1	AS2	AS3	*p*-value
Growth hormone, ng/ml	20.12 ± 1.05^b^	24.22 ± 0.98^a^	24.56 ± 1.22^a^	25.01 ± 1.32^a^	0.0486
Insulin, mlU/L	30.19 ± 1.25^c^	36.22 ± 2.23^b^	42.56 ± 1.22^a^	43.88 ± 1.65^a^	0.0239
Insulin-like growth factor-1, μg/L	130 ± 2.96^c^	138 ± 3.55^b^	149 ± 3.81^a^	153 ± 6.23^a^	0.0359

^a,b,c^Values with different small letter superscripts in the same column mean a significant difference (*p* < 0.05).

### 3.7 Effect of *Angelica sinensis* extracts on umbilical cord gene expression of piglets

**Figure 2:**
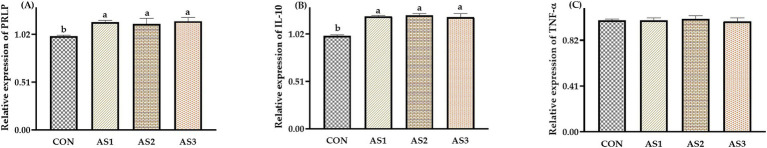
Effects of Angelica sinensis extract on umbilical cord gene expression of piglets. **(A)** The data of relative expression levels of *PRLP* (*prolactin receptor*) in the CON, AS1, AS2, and AS3 were 1.00 ± 0.01^b^, 1.15 ± 0.02^a^, 1.13 ± 0.06^a^, and 1.16 ± 0.04^a^, individually, *p* = 0.0367; **(B)** the data of relative expression levels of *IL-10* (*interleukin-10*) in the CON, AS1, AS2, and AS3 were 1.00 ± 0.01^b^, 1.21 ± 0.01^a^, 1.22 ± 0.02^a^, and 1.20 ± 0.04^a^, individually, *p* = 0.0457; **(C)** the data of relative expression levels of *TNF-α* (*tumor necrosis factor-α*) in CON, AS1, AS2, and AS3 were 1.00 ± 0.01, 1.00 ± 0.02, 1.01 ± 0.03, and 0.99 ± 0.03, individually, *p* = 0.0926. ^a,b^Values with different small letter superscripts in the same column mean a significant difference (*p* < 0.05).

The relative expressions of *PRLP* and *IL-10* were higher (*p* < 0.05) in groups treated with *Angelica sinensis* extracts than in the CON (Figure 2).

## 4 Discussion

Porcine lactation secretions serve as the gold standard nutritional matrix for neonatal swine development, delivering essential macronutrient profiles for somatic growth while simultaneously incorporating a spectrum of immunobiological factors, including immunoglobulin complexes, cytokine networks, and microbiota-shaping oligosaccharides, that orchestrate immune ontogeny, gastrointestinal barrier maturation, and symbiotic microbial colonization critical for porcine neonate viability (1, 18). Colostrum is the milk produced by mammals in the first few days after delivery, and it contains more proteins, antibodies, vitamins, and minerals than milk (3, 19). Lactoprotein is divided into casein and whey protein, with the former being the most abundant and the latter being soluble, easily digestible, and absorbable (20). Lactose is the main carbohydrate in milk, which can provide animal energy and help absorb mineral elements such as calcium, iron, and zinc (21, 22). Milk fat contains various fatty acids, which can give energy and supply fat-soluble vitamins and beneficial fatty acids such as conjugated linoleic acid for animals (20, 23). Solids-not-fat refers to the substances remaining in milk after removing water and fat, including proteins, lactose, minerals, vitamins, and other substances (20, 24). Chao et al. found that *Angelica sinensis* extracts have been used to promote breast milk secretion (25). No studies are reporting *Angelica sinensis* effect on animal milk composition. In the present study, *Angelica sinensis* extract improved lactose, milk fat, lactoprotein, and solids-not-fat contents of sows at different times after farrowing to varying degrees. The different degrees of improvement in milk composition imply that *Angelica sinensis* has a specific improvement performance on the lactation performance of sows, which may be attributed to the presence of specific bioactive compounds in *Angelica sinensis*, which are capable of augmenting the sows’ innate immune function. Consequently, such enhancement facilitates the optimization of the compositional profile and concentration of bioactive constituents within the sows’ milk (26).

Immunoglobulins are fundamental components of the animal immune system, predominantly localized in blood, tissue fluids, and exocrine secretions (27). Their principal function is to identify and bind to specific foreign antigens, including bacteria, viruses, and other pathogens, thereby initiating an immune response that either eliminates or neutralizes these invasive entities (27, 28). Interleukins are a class of proteins produced by and acting on a wide range of cells, which are essential mediators of intercellular communication in the immune system and are involved in regulating immune responses and inflammatory processes (29, 30). Tumor necrosis factors (TNFs) constitute a family of multifunctional cytokines that exert critical influence across various biological processes, including immune responses, inflammatory cascades, apoptosis, and tumor surveillance mechanisms (31, 32). Song et al. found that *Angelica sinensis* radix significantly increased serum immunoglobulin A levels in broiler chickens (33). Zhao et al. found that *Angelica sinensis* enhanced the chemical and immune barrier by expanding the lysozyme, mucin 2, sIgA, IgG, and IgM in the jejunum of Cobb 500 broilers (34). Wang et al. reported that *Angelica sinensis* polysaccharide alleviated the decline of white blood cells, lymphocytes, monocytes, neutrophils, and serum IgG and IgM caused by cyclophosphamide in rats (35). Gu et al. reported that *Angelica sinensis* polysaccharide significantly enhanced lymphocyte proliferation and improved the ratio of CD4 + to CD8 + T cells in mice (36). In this study, the *Angelica sinensis* extracts improved the milk IgA, IgG, and IgM contents in sows at different times after farrowing to varying degrees, which indicated that *Angelica sinensis* extract enhanced the immune function of Wuzhishan sows. Gong et al. found that *Angelica sinensis* significantly increased the expressions of mRNA and protein levels of interleukin-1β, interleukin-6, and tumor necrosis factor in macrophages (RAW 264.7 cells) (37). In this study, the *Angelica sinensis* extracts did not significantly improve milk levels of interleukin-6 at any period but reduced levels of tumor necrosis factor 24 h after delivery, which was inconsistent with the above study. This may be attributable to the inhibitory effects of specific bioactive compounds present in *Angelica sinensis* extracts on certain excessive inflammatory responses. Alternatively, this phenomenon could be influenced by the unique physiological or genetic characteristics inherent to the Wuzhishan sow breed.

Progesterone is an essential natural progestin that promotes the development of breast lobules and glands in preparation for lactation (38, 39). Prolactin, a protein hormone predominantly secreted by the anterior pituitary gland, plays a pivotal role in orchestrating the development and growth of the mammary glands (40). It is instrumental in initiating and maintaining lactation (40, 41). O’Cleirigh et al. found Gui Shao Di Huang Wan containing *Angelica sinensis* upregulated progesterone receptor β in Ishikawa cells (42). Chao et al. found that *Angelica sinensis* extracts increased milk production. Thus, it can be inferred that *Angelica sinensis* extracts increased prolactin levels in women (25). Liu et al. reported that Chinese herbs such as *Angelica sinensis* improved the amenorrhea caused by antipsychotics by boosting prolactin levels in women (43). In this study, the *Angelica sinensis* extracts enhanced the levels of progesterone and prolactin in the milk of sows at different stages after delivery to various degrees, which was consistent with the results of the above study, suggesting that *Angelica sinensis* extracts could regulate the lactation performance of Wuzhishan sows by regulating the levels of progesterone and prolactin in the milk. Growth hormone is a protein secreted by the pituitary gland, which plays a vital role in animal growth and development and fat metabolism (44, 45). Insulin, a peptide hormone synthesized and secreted by the pancreatic β-cells, plays a pivotal role in regulating lipid and protein metabolism while also exerting a significant influence on mineral homeostasis (46). Insulin-like growth factors (IGFs) represent a family of multifunctional regulators of cell proliferation that exert a critical influence on cell differentiation, proliferation, and overall growth and development of an organism (45, 46). Lee et al. found that *Angelica sinensis* increased the longitudinal bone growth rate in pubertal female rats by elevating growth hormone levels (47). Liu et al. reported that *Angelica sinensis* polysaccharides can improve insulin resistance in the liver by blocking receptors for advanced glycation end products in high-fat-diet and streptozotocin-induced diabetic rats (48). Liu et al. reported that *Angelica sinensis* improved insulin sensitivity through increased post-receptor insulin signaling mediated by enhancements in insulin receptor substrate-1-associated phosphatidylinositol 3-kinase step and glucose transporter subtype 4 translocations in soleus muscles of animals exhibiting insulin resistance (49). Wang et al. reported that *Angelica sinensis* polysaccharide effectively improved insulin resistance in high-fat diet-fed mice (50). Lee et al. found that *Angelica sinensis* increased local insulin-like growth factor-1 expressions in the growth plate in adolescent female rats (47). In this study, *Angelica sinensis* extracts improved the levels of growth hormone, insulin, and insulin-like growth factor-1 in the milk of sows at different stages after delivery to various degrees and also regulated the levels of all three in the serum of piglets, which was consistent with the results of the above study, indicating that *Angelica sinensis* extract may exert a regulatory influence on the lactation performance of Wuzhishan sows and the health status of their piglets through the modulation of growth hormone, insulin, and insulin-like growth factor-1 concentrations.

The *prolactin receptor* (*PRLP*) gene encodes the prolactin receptor, a transmembrane protein expressed in tissues such as mammary epithelium, liver, and lymphocytes (51). *Alpha-lactalbumin* (*LALBA*), encoded by the *LALBA* gene, interacts with various proteins, peptides, and low-molecular-weight substrates and products (52). Notably, its association with β-1,4-galactosyltransferase forms a catalytic complex that collectively facilitates the enzymatic synthesis of lactose by transferring a galactose moiety to glucose (52, 53). *Serine/threonine kinases* (*AKT1*) are a class of proteins involved in various biological processes within cells, including the cell cycle, signaling, and cellular stress responses (54). The *beta-1,4-galactosyltransferase 1* (*β4GALT1*), whose expression is increased during lactation, forms a lactose synthase complex with a-lactoprotein that catalyzes the reaction of uridine diphosphate-galactose with glucose to produce lactose (17). *Fatty acid synthase* (*FASN*) encodes a fatty acid synthase, a multi-enzyme complex responsible for synthesizing long-chain fatty acids in cells (55). The glucose transporters encoded by the GLUT1 gene constitute a family of stereospecific transmembrane proteins that facilitate the translocation of glucose across the cell membrane (56). The specific expression patterns of these transporters are pivotal in determining the cell’s glucose uptake rate (56). The *beta-casein* (*CSN2*) plays a central role in milk synthesis and can influence the composition and structure of milk proteins and the functional properties and nutritional value of dairy products (57). In this study, relative expressions of *PRLP*, *LALBA*, *AKT1*, *FASN*, *GLUT1 β4GALT1*, and *CSN2* were higher in mammary tissue in groups treated with *Angelica sinensis* extracts, which was in general agreement with the above results of milk protein, lactose, milk fat, and solids-not-fat in the milk of sows after delivery, which also implies that *Angelica sinensis* extract can regulate lactation performance of Wuzhishan sows at the genetic level. The role of *interleukin-10* (*IL-10*) in immune regulation is multifaceted; it plays a role in the maintenance of immune homeostasis as well as a critical role in the pathogenesis of many diseases (58). In this study, the relative expressions of *PRLP* and *IL-10* were higher in the umbilical cord in groups treated with *Angelica sinensis* extracts, which also implied that *Angelica sinensis* extract could modulate the immune function of Wuzhishan sows at the gene level and potentially influence the immune function of piglets.

## 5 Conclusion

This study examined the impact of incorporating *Angelica sinensis* extracts into the diet of Wuzhishan sows and revealed significant enhancements in milk composition, immune function, hormone levels, and associated gene expression across various postpartum periods. Notably, the group administered 600 mg/kg of *Angelica sinensis* extracts demonstrated superior performance in several critical areas: increased lactoprotein, lactose, milk fat, and solids-not-fat contents in milk; boosted levels of key immune indices, including IgA, IgG, and IgM; modulated the concentrations of vital hormones, such as prolactin and growth hormone; and upregulated the expression of pivotal genes in mammary tissue, namely, *PRLP*, *LALBA*, and *CSN2*. These results suggest that a dose of 600 mg/kg of *Angelica sinensis* extracts represents the optimal concentration for augmenting the lactation performance and overall health of Wuzhishan sows, thereby offering a robust theoretical foundation for its integration into Wuzhishan sow production protocols.

## Data Availability

The raw data supporting the conclusions of this article will be made available by the authors, without undue reservation.
