# Correction: Supplementation of dietary *Angelica sinensis* extracts to lactating Wuzhishan sows: effects on milk composition, immune function, milk-derived hormones, and related gene expression

**DOI:** 10.3389/fvets.2025.1648018

**Published:** 2025-07-04

**Authors:** Hongzhi Wu, Xilong Yu, Xiaoyu Zhang, Weiqi Peng, Fengjie Ji, Qian Shen, Renlong Lv

**Affiliations:** ^1^Tropical Crops Genetic Resources Research Institute, Chinese Academy of Tropical Agricultural Sciences, Haikou, China; ^2^College of Animal Science and Technology, Northeast Agricultural University, Harbin, China; ^3^College of Animal Science and Technology, Henan University of Science and Technology, Luoyang, China; ^4^Hainan Xuhuai Technology Co. Ltd., Haikou, China

**Keywords:** *Angelica sinensis* extracts, lactating sows, colostrum composition, immune function, milk hormone levels

In the published article, there was an error in the affiliations for Xilong Yu, Xiaoyu Zhang and Qian Shen, as two affiliations were omitted.

The correct authors list and affiliations list appear below:

Hongzhi Wu^1†^, Xilong Yu^2†^, Xiaoyu Zhang^3^, Weiqi Peng^1^, Fengjie Ji^1^, Qian Shen^4^, Renlong Lv^1, *^

1. Tropical Crops Genetic Resources Research Institute, Chinese Academy of Tropical Agricultural Sciences, Haikou, China

2. College of Animal Science and Technology, Northeast Agricultural University, Harbin, China.

3. College of Animal Science and Technology, Henan University of Science and Technology, Luoyang, China.

4. Hainan Xuhuai Technology Co. Ltd., Haikou, China

In the published article, the **Acknowledgements** section was not included. The section has been added, and appears below:

We extend our sincere gratitude to Ting Cao and Hanfeng Li from the Tropical Crops Genetic Resources Research Institute, Chinese Academy of Tropical Agricultural Sciences, Euphrème Ipemba and Ghislain Boungou Bakala from the National Centre for Crop Disease Control under the Ministry of Agriculture, Animal Husbandry and Fisheries of the Republic of Congo, and Lessebe Gambou Dieu Leveut from the China-aid-Congo (B) Agricultural Technology Demonstration Center of the Republic of Congo, for their valuable support in writing guidance and the provision of materials for this study.

The original article has been updated.

